# A meta-analysis of the efficacy of Roux-en-Y anastomosis and jejunal interposition after total gastrectomy

**DOI:** 10.1186/s12957-023-03002-z

**Published:** 2023-04-25

**Authors:** Yu-hang Liu, Rui Meng, Bing Zhu, Qi-qi Zhan, Xin Yang, Guan-yi Ding, Chun-liang Jia, Wei-guo Xu

**Affiliations:** 1 North China University of Science and Technology Affiliated Hospital, Tangshan, 063000 China; 2 Department of Emergency Intensive Care Unit, Yangpu Hospital, Tongji University, Shanghai, 200090 China; 3 Tangshan Gongren Hospital, Tangshan, 063000 China; 4 North China University of Science and Technology Affiliated Hospital, Tangshan, 063000 China; 5 Tangshan People’s Hospital, Tangshan, 063000 China; 6 North China University of Science and Technology Affiliated Hospital, Tangshan, 063000, China & Department of Gastrointestinal Surgery, China Hospital Medical Sciences, Shenzhen Center, No.113, Baohe District, Shenzhen, 518000 China

**Keywords:** Alimentary tract reconstruction, Jejunal interposition, Meta-analysis, Roux-en-Y, Total gastrectomy

## Abstract

**Background:**

To compare the clinical efficacy of two alimentary tract reconstruction methods—“P”-shape jejunal interposition (PJI) and Roux-en-Y anastomosis after total gastrectomy.

**Method:**

The following search phrases were utilized to search PubMed, Cochrane Library, Embase, China Academic Journals Network Full-text Database (CNKI), and Wanfang Database as of April 2022: “gastrectomy,” “Roux-en-Y,” “interposition,” “total gastrectomy,” and “jejunal interposition.” Meta-analysis of the operation time, intraoperative blood loss, complication rate, and postoperative nutritional status of patients was performed using RevMan 5.4 software.

**Results:**

A total of 24 studies and 1887 patients were included in the study. Among patients who received a total gastrectomy, the operation time in the PJI group was substantially longer than that in the Roux-en-Y group (WMD = 19.77, 95% CI: 5.84–33.70, *P* = 0.005). The incidence of postoperative reflux esophagitis in the PJI group was considerably reduced than that in the Roux-en-Y group (OR = 0.39, 95% CI: 0.28–0.56, *P* < 0.01). The probability of postoperative dumping syndrome in the PJI group was significantly lower than that in the Roux-en-Y group (OR = 0.27, 95% CI: 0.17–0.43, *P* < 0.01), and the postoperative body mass changes were significantly lower in the PJI group than in the Roux-en-Y group (WMD = 3.94, 95% CI: 2.24–5.64, *P* < 0.01). The PJI group had substantially higher postoperative hemoglobin, albumin, and total protein levels than the Roux-en-Y group (WMD = 13.94, 95% CI: 7.77–19.20, *P* < 0.01; WMD = 3.97, 95% CI: 2.58–5.37, *P* < 0.01; WMD = 5.31, 95% CI: 3.45–7.16, *P* < 0.01). The prognostic nutritional index was higher in the PJI group than in the Roux-en-Y group (WMD = 9.25, 95% CI: 7.37–11.13, *P* < 0.01).

**Conclusion:**

PJI is a safe and effective reconstruction method and is superior to Roux-en-Y anastomosis in the prevention and treatment of postoperative complications and postoperative nutritional recovery in patients after total gastrectomy.

**Supplementary Information:**

The online version contains supplementary material available at 10.1186/s12957-023-03002-z.

## Background

Gastric carcinoma is a highly prevalent malignant tumor in clinical practice, with a high invasiveness and metastatic rate; it is the fifth most common cancer in the world [1]. Its morbidity and mortality rate rank second and third, respectively, in China [2]. With the improvements made in laparoscopic technology, laparoscopic total gastrectomy (LTG) is the procedure of choice for the treatment of advanced gastric cancer. However, the choice of alimentary tract reconstruction method following total gastrectomy depends on the postoperative recovery and long-term life quality of patients and has traditionally been the focus of clinical research [3]. Complications associated with total gastrectomy, including reflux esophagitis, anastomotic leakage, anastomotic hemorrhage, and anastomotic stenosis, are the primary factors limiting patient recovery following surgery and may even lead to unplanned repeat surgery or death [4]. There are numerous methods for reconstructing the alimentary tract after a total gastrectomy [5], and each procedure has its own advantages. However, a consensus has not yet been reached as to which method is superior [6]. Using meta-analysis, we examined the optimal surgical technique for alimentary tract reconstruction following total gastrectomy based on the literature on the most prevalent methods utilized in clinical practice (esophageal jejunal Roux-en-Y anastomosis and jejunal interposition). In esophageal jejunal Roux-en-Y anastomosis [6], the duodenal stump was closed, the lower end of the esophagus was anastomosed with the distal jejunum, and the continuous broken end of the duodenum was anastomosed with the output end of the jejunum in y-style. Jejunal interposition is more common in China but is not very common in the West. The interleaved jejunum is a section of jejunum used in the operation. The upper end is anastomosed with the esophagus, and the lower end is anastomosed with the pancreatoduodenum. In the middle of the jejunum, the anastomosis is opened to the postoperative residual stomach. This is equivalent to the establishment of a jejunal tee between the esophagus, the remnant stomach, and the intestine. Intermediate jejunum has a certain food storage function [6].

## Data and method

### Literature search strategy

As of April 2022, we searched the published literature on alimentary tract reconstruction following total gastrectomy on PubMed, Cochrane Library, Embase, China Academic Journals Network Full-text Database (CNKI), and Wanfang Database. In addition, we conducted an exhaustive search based on the reference list. The language of the search was not limited. The search terms were “gastrectomy,” “Roux-en-Y,” “interposition,” “total gastrectomy,” and “jejunal interposition.”

### Inclusion and exclusion criteria

#### Inclusion criteria

(1) The study type was set as a randomized controlled trial (RCT); (2) the included participants were all gastric cancer patients who underwent selective total gastrectomy and alimentary reconstruction—either Roux-en-Y anastomosis or jejunal interposition; (3) none of the patients underwent treatments such as radiotherapy or chemotherapy prior to surgery, which could have affected the outcome; (4) the literature compared Roux-en-Y and “P”-shape jejunal interposition (PJI); (5) the study should have close follow-up and detailed clinical data, and the endpoint event should be derivable from the original data.

#### Exclusion criteria

(1) Reviews, expert recommendations, meta-analysis, case reports, animal experiments, and cadavers; (2) few cases included in any group of the literature (< 10 cases); (3) patients with distant metastases, recurrent tumors, or other systemic malignancies; (4) literature that did not have any outcome indicators; (5) there were obvious differences in postoperative treatments; (6) repetitive literature; (7) literature where the original text could not be retrieved.

### Outcome indicators

The outcome indicators mainly included the occurrence of postoperative complications and nutritional indicators of patients.

### Literature screening and data extraction

According to the defined inclusion and exclusion criteria, two independent reviewers separately evaluated the literature, and a senior investigator made the final determination in cases of disagreement. Two individuals independently appraised the extracted literature, and the pertinent data mostly consisted of study characteristics, basic patient data, surgery-related outcome indicators, postoperative complication outcome index, and postoperative nutrition index.

### Literature quality evaluation

The literature was independently evaluated by two researchers and the quality assessment of RCTs was typically completed using the Risk of Bias tool developed by the Cochrane Collaboration.

### Statistical analysis

Statistical analysis was performed using RevMan 5.4 software. The mean difference (MD) was used as the effect index for the measurement data, and the odds ratio (OR) was used as the effect index for the counting data. The point estimate and 95% CI were calculated for each effect size. The chi-square test was used to determine the heterogeneity between the results of the study (test level was *α* = 0.1). Also, the heterogeneity was determined by the combination of the *I*^2^ value. For studies with no statistical heterogeneity, the fixed-effect model was adopted for pooled analysis. For studies with statistical heterogeneity, the random-effects model was adopted for pooled analysis. Significant clinical heterogeneity was processed using methods such as subgroup analysis or sensitivity analysis, or only descriptive analysis. The test level for meta-analysis was *α* = 0.05.

## Results

### Literature search results

The preliminary screening yielded a total of 498 articles, including 185 in English and 313 in Chinese. By examining the titles and abstracts, 460 irrelevant and non-RCT references were excluded, and 38 RCTs were initially included. After a comprehensive reading of the full text, 24 articles were included in the study, including 4 in English and 20 in Chinese. In the 24 included RCTs, there were a total of 1887 cases of alimentary tract reconstruction following total gastrectomy for gastric cancer, including 944 cases in the “P”-shape jejunal interposition (PJI) group and 943 cases in the Roux-en-Y group (Supplemental Fig. 1). Randomization and double-blinded allocation were adopted in the included studies. All patients in the 24 studies were followed up after treatment, and loss of follow-up was noticed in 2 studies. The Jadad scores were used for quality assessment of the RCTs, and the scores of the 24 included studies were all above 4, indicating high quality (Table 1).

**Fig. 1:**
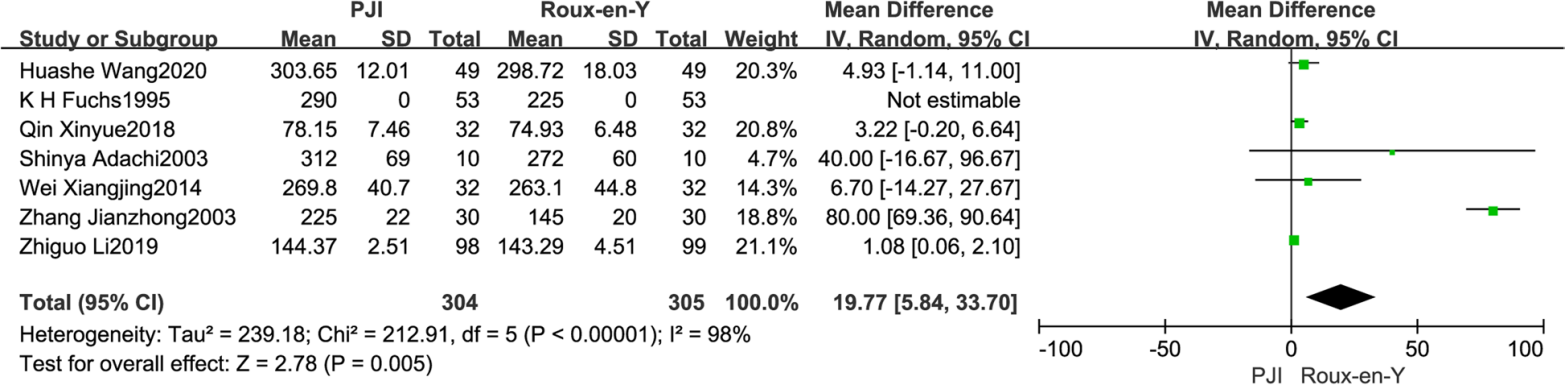
Comparison of operation time between the PJI group and the Roux-en-Y group

**Table 1:** Basic characteristics and quality evaluation of the included literature

Literature	Period	Cases		Research center	Randomization	Main outcome indicators^α^	Allocation concealment	Integrity
			Jejunal interposition					
Fuchs et al. [7]	1985–1990	53	53	Multi-center	Yes	1	Yes	Yes
Adachi et al. [8]	1995–1996	10	10	Single-center	Yes	1, 2, 4	Yes	Yes
Zhang et al. [9]	1998–2001	30	30	Single-center	Yes	1, 3, 5, 6, 7	Yes	Yes
Liu et al. [10]	1995–2001	52	42	Single-center	Yes	3, 4, 5, 6	Yes	Yes
Xu et al. [11]	2004–2005	16	17	Single-center	Yes	5, 6	Yes	Yes
Yang et al. [12]	2002–2004	15	16	Single-center	Yes	5, 6	Yes	Yes
Dong et al.[13]	2005–2007	32	32	Single-center	Yes	3, 4, 5, 6	Yes	Yes
Long et al.[14]	2009–2011	38	38	Single-center	Yes	3, 4, 6	Yes	Yes
Zhan et al.[15]	2012–2013	57	52	Single-center	Yes	3, 4, 5, 6	Yes	Yes
Zhang et al.[16]	2010–2011	41	41	Single-center	Yes	3, 6	Yes	Yes
Chen et al.[17]	2010–2013	25	25	Single-center	Yes	2, 3, 4, 5	Yes	Yes
Wei et al.[18]	2008–2013	32	32	Single-center	Yes	1, 2, 3, 4	Yes	Yes
Shao et al.[19]	2012–2013	32	32	Single-center	Yes	3, 4, 5, 6	Yes	Yes
Jiang et al.[20]	2015	48	48	Single-center	Yes	3, 4, 5, 6	Yes	Yes
Tao et al.[21]	2013–2016	38	39	Single-center	Yes	2, 3, 4, 5, 6	Yes	Yes
Zhou et al.[22]	2014–2016	49	49	Single-center	Yes	3, 4, 6	Yes	Yes
Li et al.[23]	2015–2016	42	42	Single-center	Yes	3, 5, 6	Yes	Yes
Qin et al.[24]	2014–2016	32	32	Single-center	Yes	1, 2, 3, 4, 6	Yes	Yes
Tao et al.[25]	2016–2017	38	39	Single-center	Yes	2, 4, 5, 6	Yes	Yes
Yang et al.[26]	2012–2015	30	40	Single-center	Yes	6	Yes	Yes
Li et al.[27]	2015–2017	99	98	Single-center	Yes	1, 2, 3, 5, 6	Yes	Yes
Liu et al.[28]	2017	30	30	Single-center	Yes	3, 4, 5, 6	Yes	Yes
Wang et al.[29]	2012–2017	55	58	Multi-center	Yes	1, 2, 3, 6	Yes	Yes
Li et al.[30]	2017–2019	49	48	Single-center	Yes	2, 3, 4, 5, 6, 7	Yes	Yes

Note: ^α^main indicators: 1, operation time; 2, intraoperative blood loss; 3, incidence of reflux esophagitis; 4, incidence of dumping syndrome; 5, changes in body mass; 6, changes in postoperative hemoglobin, albumin, and total protein levels; 7, prognostic nutritional index (PNI = serum albumin value ALB: g/L + 5 × total number of peripheral blood lymphocytes TLC: 10^9^/L)

### Efficacy analysis

#### Operation time

Heterogeneity of operation time between the two surgical methods was reported in 7 studies (*P* < *0.01*,* I*^*2*^ = *98%*) [7–9, 18, 24, 27, 29]. The random-effects model was adopted for analysis, and the results showed that the operation time in the PJI group was significantly higher than that in the Roux-en-Y group (WMD = 19.77, 95% CI: 5.84–33.70, *P* = *0.005*) (Fig. 1).

#### Intraoperative blood loss

**Fig. 2:**
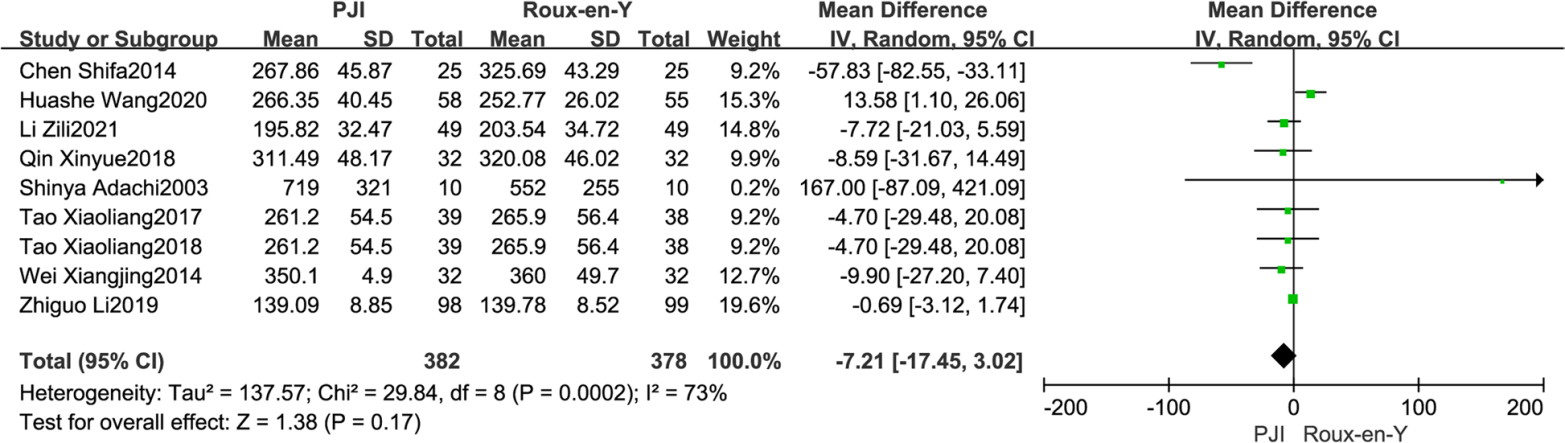
Comparison of intraoperative blood loss between the PJI group and the Roux-en-Y group

Intraoperative blood loss was reported in 9 included studies [8, 17, 18, 21, 24, 25, 27, 29, 30]. Heterogeneity was noticed between the studies (*P* = 0.002, *I*^2^ = 73%), and the random-effects model was adopted for analysis. The results showed that there was no significant difference in intraoperative blood loss between the two groups (WMD =  − 7.21, 95% CI =  − 17.45–3.02,* P* = 0.17) (Fig. 2).

#### Reflux esophagitis

**Fig. 3:**
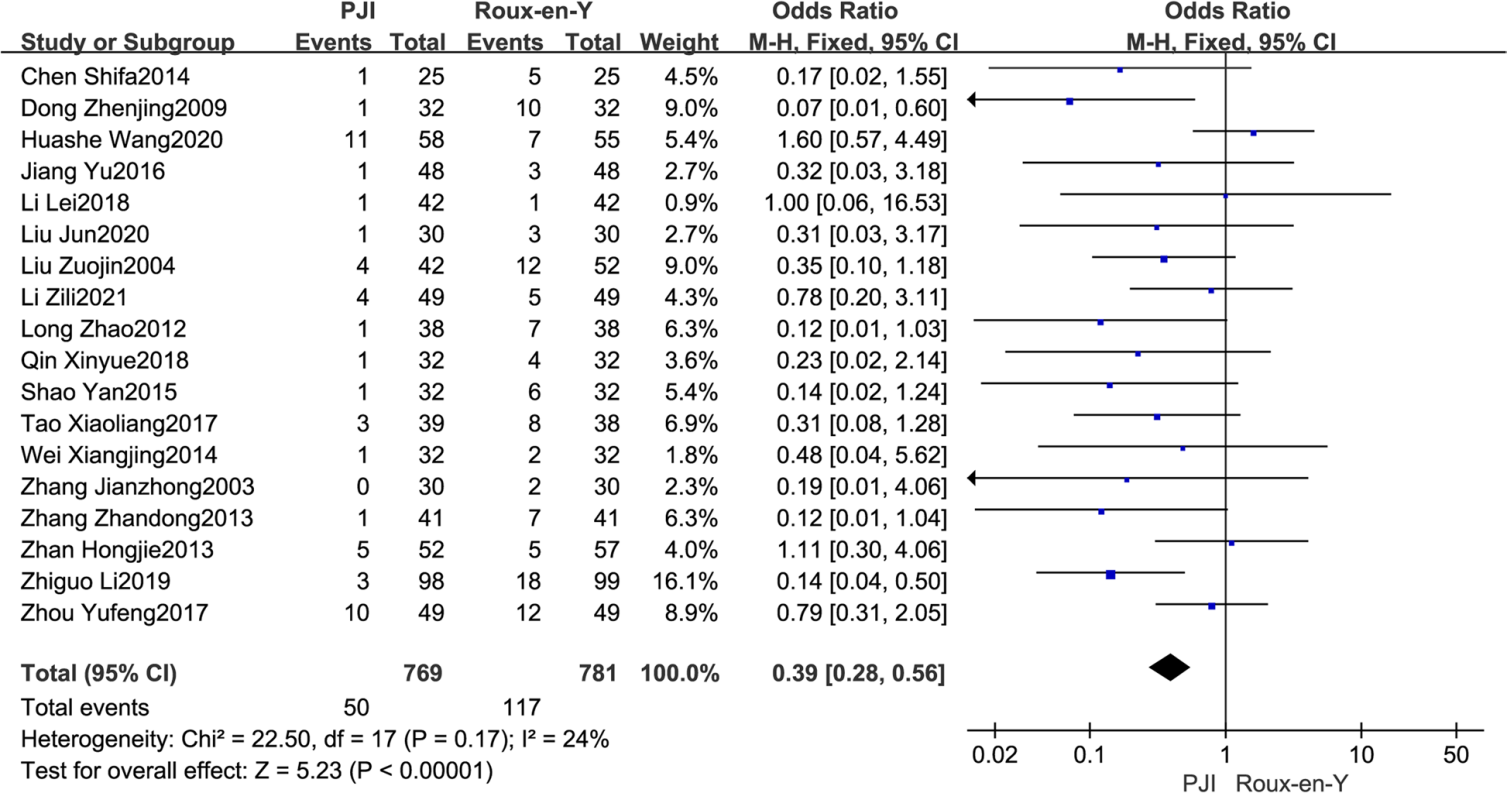
Comparison of the incidence of postoperative reflux esophagitis between the PJI group and the Roux-en-Y group

The occurrence of postoperative reflux esophagitis in both groups was reported in 18 studies [9, 10, 13–24, 27–30]. There was no heterogeneity between studies (*P* = 0.17, *I*^2^ = 24%), and the fixed−effect model was used for analysis. The results revealed that the incidence of postoperative reflux esophagitis in the PJI group was significantly lower than that in the Roux-en-Y group (OR = 0.39, 95% CI: 0.28–0.56, *P* < *0.01*) (Fig. 3).

#### Dumping syndrome

**Fig. 4:**
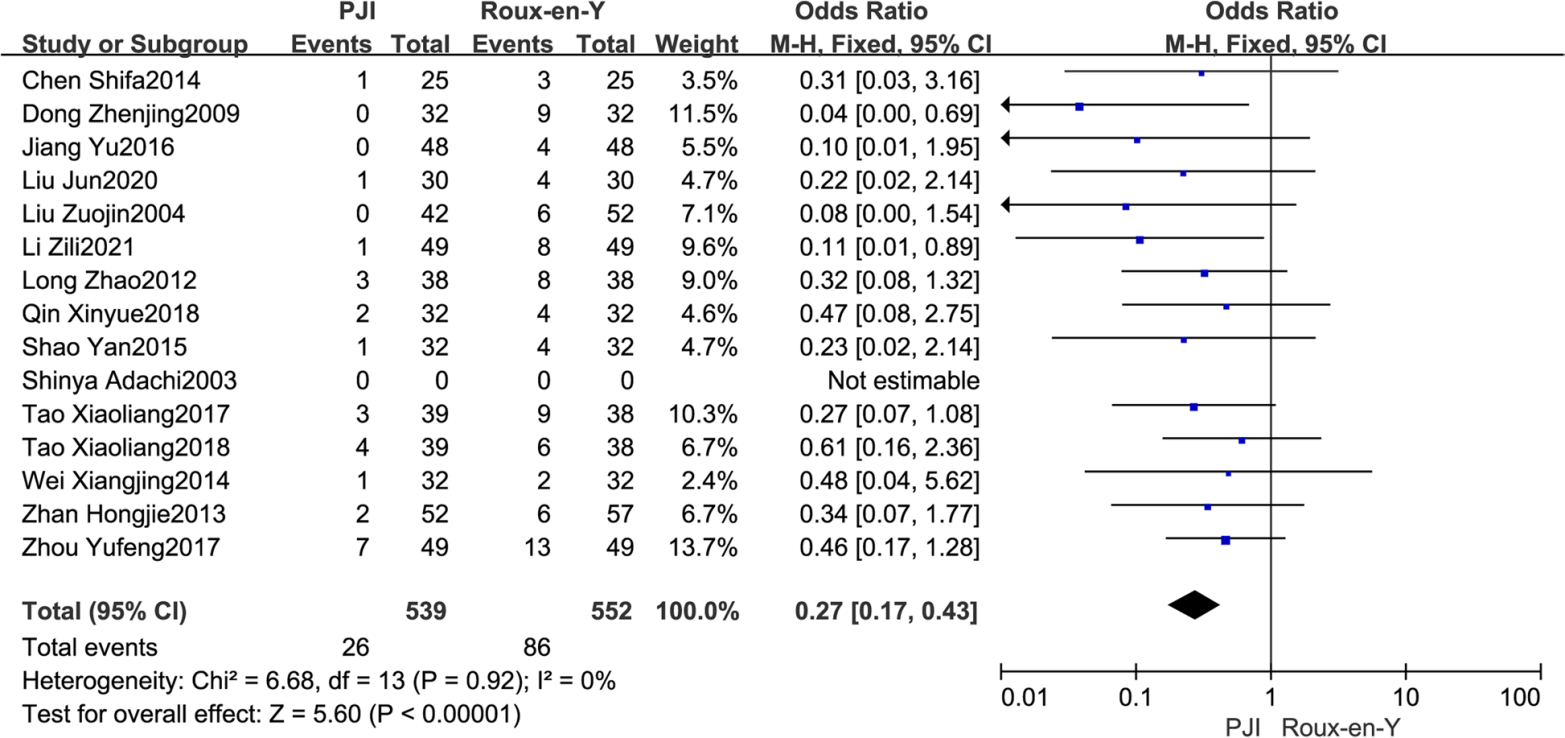
Comparison of the incidence of postoperative dumping syndrome between the PJI group and the Roux-en-Y group

The occurrence of postoperative dumping syndrome in both groups was reported in 14 studies [10, 13–15, 17–22, 24, 25, 28, 30]. There was no heterogeneity between the studies (*P* = 0.92, *I*^2^ = 0), and the fixed−effect model was adopted for pooled analysis. The results revealed that the probability of postoperative dumping syndrome in the PJI group was significantly lower than that in the Roux-en-Y group (OR = 0.27, 95% CI: 0.17–0.43, *P* < *0.01*) (Fig. 4).

#### Changes in postoperative body mass

**Fig. 5:**
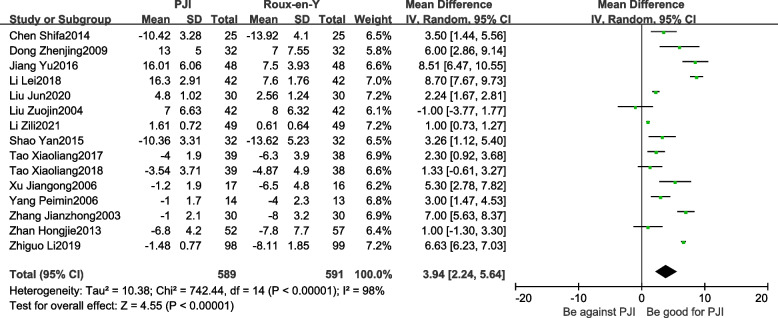
Comparison of changes in postoperative body mass between the PJI group and the Roux-en-Y group

Changes in postoperative body mass in both groups were reported in 15 studies [9–13, 15, 17, 19–21, 23, 25, 27, 28, 30]. There was heterogeneity between the studies (*P* < 0.01, *I*^2^ = 98%), and the random−effects model was adopted for analysis. The results revealed that the changes in postoperative body mass in patients in the PJI group were significantly lower than those in the Roux-en-Y group (WMD = 3.94, 95% CI: 2.24–5.64, *P* < *0.01*) (Fig. 5).

#### Changes in postoperative hemoglobin, albumin, and total protein

**Fig. 6:**
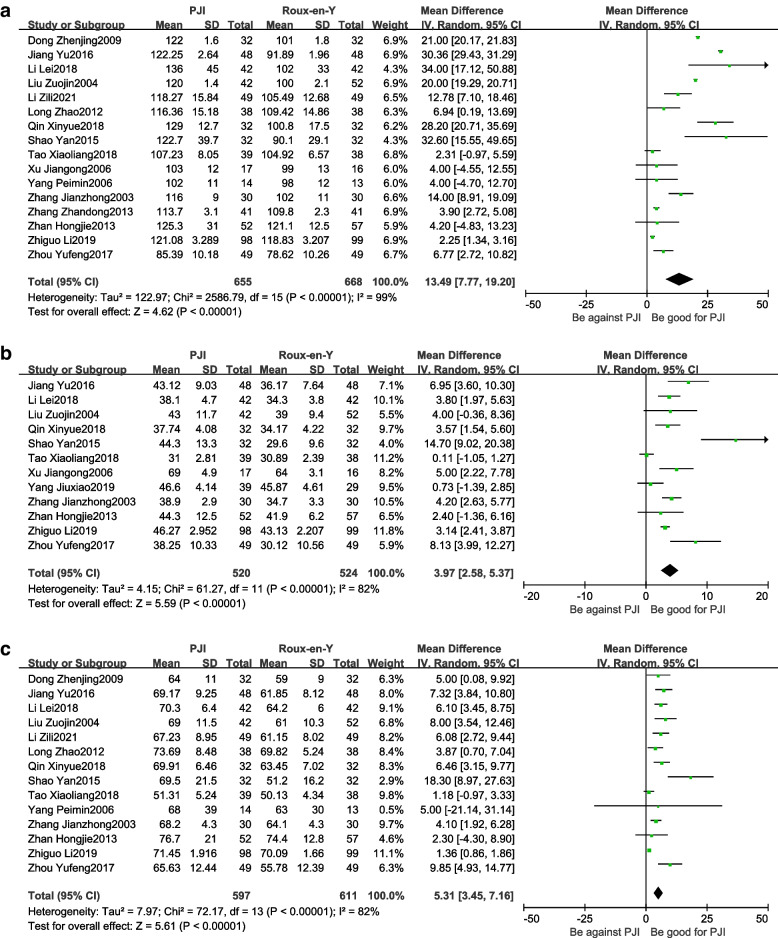
Comparison of changes in postoperative hemoglobin, albumin, and total protein levels between the PJI group and the Roux-en-Y group. **A** Comparison of postoperative hemoglobin between the PJI group and the Roux-en-Y group. **B** Comparison of postoperative albumin between the PJI group and the Roux-en-Y group. **C** Comparison of postoperative total protein between the PJI group and the Roux-en-Y group

- (1) Changes in postoperative hemoglobin of patients in the two groups were reported in 16 studies [9–16, 19, 20, 22–25, 27, 30]. There was heterogeneity between the studies (*P* < 0.01, *I*^2^ = 99%), and the random−effects model was adopted for analysis. The results revealed that the postoperative hemoglobin of patients in the PJI group was higher than that in the Roux-en-Y group (WMD = 13.94, 95% CI: 7.77–19.20, *P* < *0.01*) (Fig. 6A).
- (2) Postoperative albumin of patients in the two groups was reported in 12 studies [9–11, 15, 19, 20, 22–27]. There was heterogeneity between the studies (*P* < 0.01, *I*^2^ = 82%), and the random-effects model was adopted for analysis. The results showed that postoperative albumin in the PJI group was higher than that in the Roux-en-Y group (WMD = 3.97, 95% CI: 2.58–5.37,* P* < 0.01) (Fig. 6B).
- (3) Postoperative total protein of patients in the two groups was reported in 14 studies [9, 10, 12–15, 19, 20, 22–25, 27, 30]. There was heterogeneity between the studies (*P* < 0.01, *I*^2^ = 82%), and the random−effects model was adopted for analysis. The results revealed that postoperative total protein in the PJI group was higher than that in the Roux-en-Y group (WMD = 5.31, 95% CI: 3.45–7.16, *P* < *0.01*) (Fig. 6C).

#### Prognostic nutritional index

**Fig. 7:**
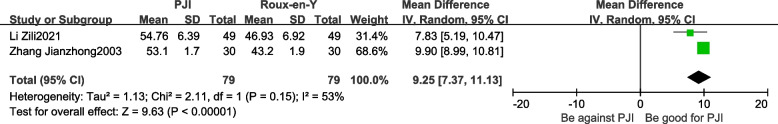
Comparison of the postoperative prognostic nutritional index between the PJI group and the Roux-en-Y group

Prognostic nutritional index of patients in the two groups was reported in 2 studies [9, 30]. There was heterogeneity between the studies (*P* < 0.15, *I*^2^ = 53%), and the random−effects model was adopted for analysis. The results revealed that the prognostic nutritional index of patients in the PJI group was better than that in the Roux−en-Y group (WMD = 9.25, 95% CI: 7.37–11.13, *P* < *0.01*) (Fig. 7).

### Publication bias

**Fig. 8:**
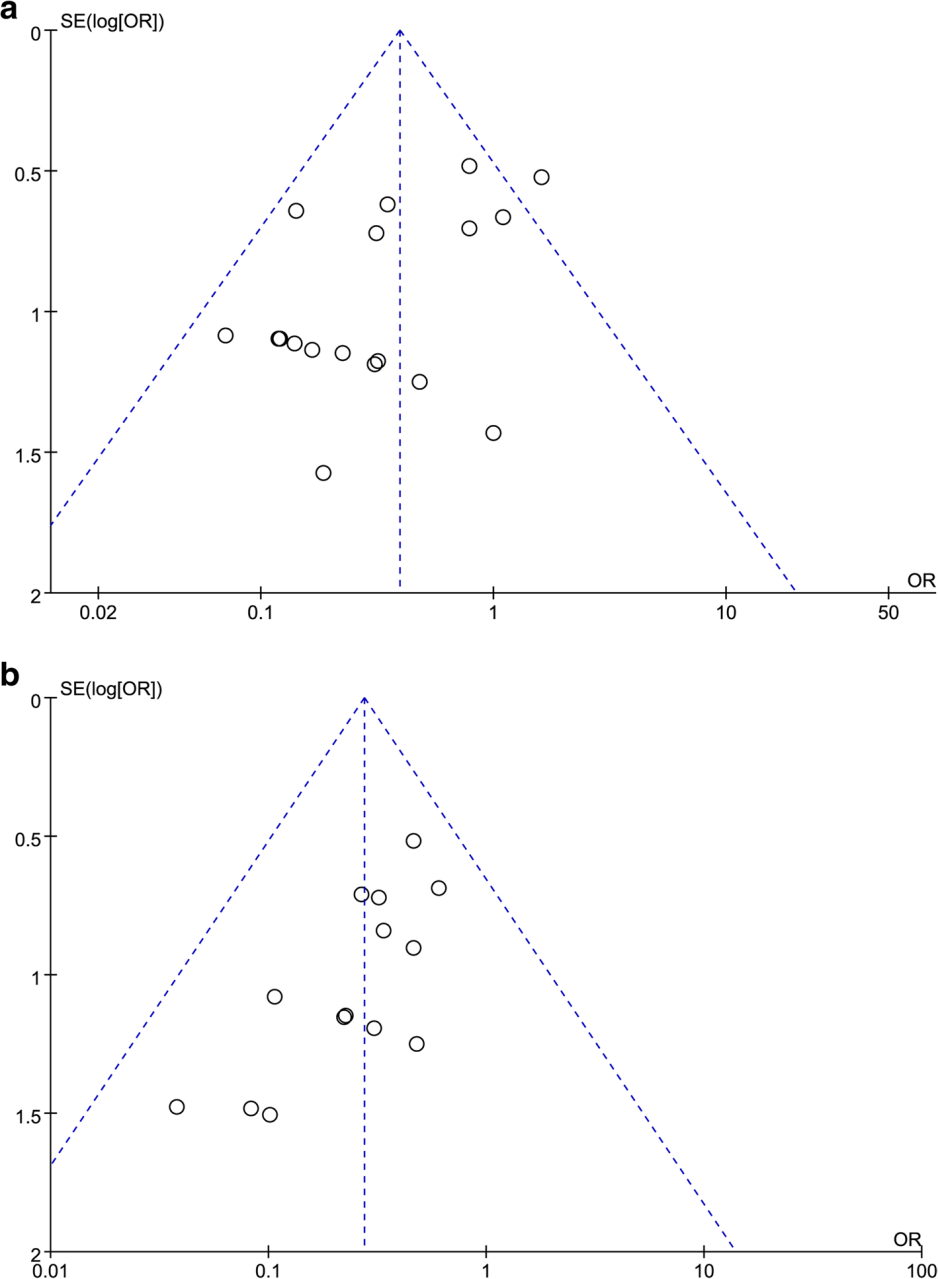
Funnel plots of the incidences of reflux esophagitis and dumping syndrome. **A** Funnel plot of the publication bias related to reflux esophagitis. **B** Funnel plot of the publication bias related to dumping syndrome

The funnel plots of the incidences of reflux esophagitis and dumping syndrome were symmetrical, indicating a small publication bias (Fig. 8).

## Discussion

Gastric cancer is one of the most prevalent malignant tumors in China. As a result of dietary and lifestyle changes, the incidence of gastric cancer is increasing annually, the affected population is getting younger, [31] and the mortality rate is approximately 75%. The current standard treatment method for advanced gastric cancer is total gastrectomy, which has been performed for more than a century. After a total gastrectomy, the patient may experience loss of appetite or decreased food intake, resulting in severe malnutrition. Consequently, the reconstruction of the alimentary tract following a total gastrectomy is a major concern. Currently, there is no consensus on the alimentary tract reconstruction method following total gastrectomy. The traditional esophageal jejunal Roux-en-Y anastomosis is often preferred due to its simple procedure and effectiveness in improving postoperative reflux. The complications after esophageal jejunal Roux-en-Y anastomosis include anastomotic leakage, empyema, subdiaphragmatic abscess, and postoperative intestinal obstruction. However, the issue of nutrient absorption after total gastrectomy is a matter of concern. To improve the long-term life quality of patients, jejunal interposition that preserves the duodenum is preferred. Roux-en-Y anastomosis transected jejunum, which damaged the integrity of conduction between the intestinal tract and related nerves, and food did not pass through the duodenum, which interfered with normal digestion and absorption function. Compared with the traditional Roux-en-Y anastomosis, the interposition jejunal gastroreplacement operation did not completely cut the jejunum during the operation, so as to maintain the integrity of the patient’s intestinal physiology and nerve conduction, and to preserve the patient’s digestive tract function to the greatest extent, so as to avoid postoperative reflux esophagitis. The physiological function of the duodenum is preserved, which is conducive to promoting the secretion of cholecystokinin and pancreatic fluid, and provides a good intestinal alkaline environment for patients, which effectively reduces the occurrence probability of retention syndrome and anastomotic leakage, and also plays an important role in the growth of intestinal mucosa. This operation can effectively improve the quality of life of patients after surgery. However, jejunal interposition is a relatively complex procedure, involves a longer operation time, and correspondingly has an increased risk of postoperative complications. Its complications include anastomotic leakage, anastomotic hemorrhage, anastomotic stenosis, intestinal obstruction, infection, obstruction of jejunal bag emptying, reflux esophagitis, R-S syndrome, dumping syndrome, early fullness, gallstones, bile reflux, Roux-en-Y retention syndrome, anemia, malnutrition, etc. Although there are several controversies, a vast number of clinical studies have been conducted on this topic. However, there is a lack of large-scale, multi-center, prospective RCTs. Publication bias was frequently observed in studies, and there is still no consensus on the reconstruction method of the alimentary tract following total gastrectomy.

The results of this study revealed that compared with Roux-en-Y anastomosis, patients who underwent jejunal interposition after total gastrectomy had lower incidence of reflux esophagitis and dumping syndrome; higher prognostic nutritional index, postoperative hemoglobin, albumin, and total protein levels; and less changes in postoperative body mass; however, the operation time was relatively longer in the jejunal interposition group. There was no significant difference in intraoperative blood loss between the two groups.

However, one thing that must be brought to our attention is that jejunal interposition is usually a three-anastomoses technique, which potentially increases the overall risk of leakage and other anastomotic complications when compared to a two-anastomoses technique such as the Roux-en-Y. So we should pay more attention to this point in clinical practice and in future studies.

There are certain limitations to this study. First, the sample sizes of some of the studies included in this meta-analysis were small, which may have affected the results. Second, the research was conducted in a single center and there were insufficient measurement indicators in some of the included studies, which may have affected the strength of the results. Third, there may be a distribution bias as studies in other languages were not included. Fourth, the vast majority of the studies in this field come from China and its respective patient population, which could cause bias. Finally, with the development of total gastrectomy and the rapid adoption of laparoscopic techniques, traditional open surgery and laparoscopic surgery may have also had an impact on the outcome.

## Conclusion

In conclusion, compared with Roux-en-Y anastomosis, jejunal interposition not only effectively prevents postoperative complications, but also has significant advantages in improving long-term prognosis and life quality of patients, making it a safe and effective method for reconstructing the alimentary tract. However, there is still no consensus on how to reconstruct the alimentary tract following total gastrectomy, and there is a need for a substantial number of clinical cases to resolve the same.

## Supplementary Information

**Additional file 1:** **Supplemental Fig. 1.** A Total gastrectomy Roux-en-Y anastomosis. The duodenal stump was first closed, and the jejunum was severed 15-20 cm below the Treitz ligament. End-to-side anastomosis was performed between the distal jejunum and the esophagus, and the stump was kept 3-5 cm and closed. End-to-side jejunal-jejunal anastomosis was performed 40 cm from the distal end of the esophagojejunal anastomosis. B: Total gastrectomy with jejunostomy. Firstly, end-to-side esophagojejunal anastomosis was performed 40 cm below the Treitz ligament, and end-to-side anastomosis was performed on the duodenum at 35 cm away from the anastomosis, and side-to-side anastomosis was performed on the jejunoduodenum about 5 cm below the jejunoduodenal anastomosis and 20 cm below the Treitz ligament. The input branch segment was 5-7 cm away from the esophagojejunoduodenal anastomosis and the output branch segment was 2 cm away from the distal end of the jejunoduodenal anastomosis.

## Data Availability

The datasets used and/or analyzed during the current study are available from the corresponding author on reasonable request.

## Abbreviations

LTG: Laparoscopic total gastrectomy
MD: Mean difference
OR: Odds ratio
PJI: “P”-shape jejunal interposition

## References

[1] Thrift AP, Nguyen TH (2021). Gastric cancer epidemiology. Gastrointest Endosc Clin N Am.

[2] Guidelines for diagnosis and treatment of gastric cancer (2018 Edition) [J]. J Multidiscip Cancer Manag. 2019;5(01):55–82.

[3] Du HY, Wang ZJ, Yang HT (2019). Advances research on digestive tract reconstruction in totally laparoscopic total gastrectomy [J]. Medical Journal of National Defending Forces in Northwest China..

[4] Huang CM, Ji JF, Qin XY (2021). Chinese expert consensus on the prevention and treatment of esophagojejunostomy complications after total gastrectomy for gastric cancer (2020 edition). Chin J Pract Surg.

[5] Cai QP (2021). Reasonable selection of digestive tract reconstruction methods after total gastrectomy[J]. Shanghai Yi Yao..

[6] Li LP, Cui HP, Shang L (2021). Choice and consideration of digestive tract reconstruction after total gastrectomy and proximal gastrectomy. Chin J Digest Surg.

[7] Fuchs KH, Thiede A, Engemann R, Deltz E, Stremme O, Hamelmann H (1995). Reconstruction of the food passage after total gastrectomy: randomized trial. World J Surg.

[8] Adachi S, Inagawa S, Enomoto T, Shinozaki E, Oda T, Kawamoto T (2003). Subjective and functional results after total gastrectomy: prospective study for longterm comparison of reconstruction procedures. Gastric Cancer.

[9] Zhang JZ, Lu HS, Wu XY, Huang CM, Wang C, Guan GX, Zhang XF (2003). Influence of different procedures of alimentary tract reconstruction after total gastrectomy for gastric cancer on the nutrition and metabolism of patients: a prospective clinical study. Natl Med J Chin.

[10] Liu ZJ, Luo YS, Ge HY, Ma XG, Wang LM, Cheng YD, Zhang CJ (2004). Influence of jejunal interposition pouch reconstruction on nutritional condition of patients after total gastrectomy. Chin J Bases Clin Gen Surg.

[11] Xu JG (2006). Clinical comparison of two types of total gastrectomy for gastric replacement. Pract Clin Med..

[12] Yang PM (2006). Comparison of two gastric replacement methods after total gastrectomy. Chin J Gastroenterol.

[13] Dong ZJ, Wu LX, Li EJ (2009). Study on reconstruction of digestive tract after total gastrectomy for gastric cancer. Hebei Med.

[14] Long Z (2012). Observation on clinical efficacy of digestive tract reconstruction after total gastrectomy. Chin Med Sci.

[15] Zhan HJ. Prospective randomized controlled clinical study of functional jejunum interposition after total gastrectomy [D]. Tianjin Medical University; 2013.

[16] Zhang ZD, Ma F, Zhang YL, Ma EM, Kong Y, Liu HX, Hua YW (2013). Comparison study of three methods of digestive tract reconstruction after radical gastrectomy in gastric cancer patients. Chin J Gastrointes Surg.

[17] Chen SF (2014). Observation on the therapeutic effect of modified jejunal interposition for gastric replacement on reconstruction of digestive tract after total gastrectomy for gastric cancer. Med Theory Pract.

[18] Wei XJ (2014). Clinical observation on total gastrectomy with OItype jejunal interposition in situ generation for gastric cancer. Guangxi Med.

[19] Shao Y, Yin L (2015). Evaluation of P shape jejunum pan jejunum esophagus Royx-en-Y anastomosis and modified jejunal interposition in total gastrectomy for gastric cancer. Chin J Clin Oncol Rehabil.

[20] Jiang Y, Zheng MS, Wu XL, Lin YF (2016). Effect of interposition jejunum reconstruction on nutritional metabolism and gastrointestinal function of patients after total gastrectomy. Chin Health Standard Manag.

[21] Tao XL, Jiang HW, Xu L, Chen J, Xiao SM, Zhou HY (2017). Observation on the effect of adjusting double channel jejunum interposition for gastric reconstruction after total gastrectomy. Shandong Med.

[22] Zhou FY (2017). Comparison of different digestive tract reconstruction methods in patients undergoing total gastrectomy for gastric cancer [J]. Chin Med Guide.

[23] Li L (2018). The effect of jejunal interposition and Roux-en-Y anastomosis on gastric cancer patients after radical total gastrectomy. Modern Diag Treatment.

[24] Qin XY (2018). Comparison of functional jejunum interposition and P-Roux-en-Y jejunum interposition in gastric cancer patients after radical total gastrectomy. Henan Med Res.

[25] Tao XL. Early efficacy evaluation of regulated double channel jejunal interposition for gastric cancer after total gastrectomy [D]. Southwest Medical University; 2018.

[26] Yang JX, Jiang HW, Xu L, Tao XL, Zhou HY, Wang ZH (2019). Changes of serum copper in patients with different digestive tract reconstruction after total gastrectomy for gastric cancer. Chin J Clin Res.

[27] Li Z, Dong J, Huang Q, Zhang W, Tao K (2019). Comparison of three digestive tract reconstruction methods for the treatment of Siewert II and III adenocarcinoma of esophagogastric junction: a prospective, randomized controlled study. World J Surg Oncol.

[28] Liu J, Liu XW (2020). Effects of two different reconstruction methods of digestive tract after radical total gastrectomy on quality of life and immune nutrition status in patients with gastric cancer. Contemp Med.

[29] Wang H, Hu X, Chen S, Xiang J, Yang Z, Zhou Z, Chen Y, Lin Y, Chen Y, Peng J (2020). Functional jejunal interposition versus Roux-en-Y anastomosis after total gastrectomy for gastric cancer: a prospective randomized clinical trial. Surg Oncol.

[30] Li ZL, Xie XY (2021). The effects of different digestive tract reconstruction methods on gallbladder contraction function and postoperative complications in patients with gastric cancer after laparoscopic total gastrectomy. Pract J Clin Med.

[31] Liu YW, Cheng N, Yang DZ, Jia Y, Dong M, Tang X, Yang XD, Yuan HB (2022). Effects of different reconstruction and anastomosis methods of digestive tract on patients with gastric cancer after total gastrectomy. Cancer Clin Rehabil Chin.

