# Supplementary figures and images for: A meta-analysis of the efficacy of Roux-en-Y anastomosis and jejunal interposition after total gastrectomy
Source: World J Surg Oncol. 2023 Apr 25;21:136. doi: 10.1186/s12957-023-03002-z (PMC10127366; doi:10.1186/s12957-023-03002-z)

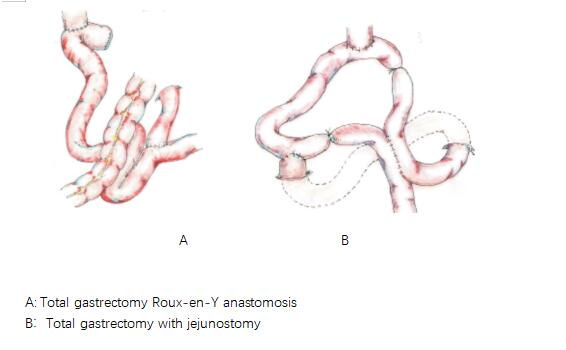

Supplement: Supplementary file 1 — Additional file 1: Supplemental Fig. 1. A Total gastrectomy Roux-en-Y anastomosis. The duodenal stump was first closed, and the jejunum was severed 15-20 cm below the Treitz ligament. End-to-side anastomosis was performed between the distal jejunum and the esophagus, and the stump was kept 3-5 cm and closed. End-to-side jejunal-jejunal anastomosis was performed 40 cm from the distal end of the esophagojejunal anastomosis. B: Total gastrectomy with jejunostomy. Firstly, end-to-side esophagojejunal anastomosis was performed 40 cm below the Treitz ligament, and end-to-side anastomosis was performed on the duodenum at 35 cm away from the anastomosis, and side-to-side anastomosis was performed on the jejunoduodenum about 5 cm below the jejunoduodenal anastomosis and 20 cm below the Treitz ligament. The input branch segment was 5-7 cm away from the esophagojejunoduodenal anastomosis and the output branch segment was 2 cm away from the distal end of the jejunoduodenal anastomosis. [file 12957_2023_3002_MOESM1_ESM.jpg]
